# From oesophageal cancer to constrictive pericarditis: oesophagopericardial fistula complicated by *Streptococcus equi* infection—a case report

**DOI:** 10.1093/ehjcr/ytag700

**Published:** 2026-09-15

**Authors:** Sofia Esteves, Ana Rita Figueiredo, Joana Rigueira, Catarina Sousa, Fausto J Pinto

**Affiliations:** Department of Cardiology, Unidade Local de Saúde de Santa Maria (ULSSM), CAML, CCUL@RISE, Faculdade de Medicina, Universidade de Lisboa, Avenida Professor Egas Moniz MB, 1649-028 Lisboa, Portugal; Department of Cardiology, Unidade Local de Saúde de Santa Maria (ULSSM), CAML, CCUL@RISE, Faculdade de Medicina, Universidade de Lisboa, Avenida Professor Egas Moniz MB, 1649-028 Lisboa, Portugal; Department of Cardiology, Unidade Local de Saúde de Santa Maria (ULSSM), CAML, CCUL@RISE, Faculdade de Medicina, Universidade de Lisboa, Avenida Professor Egas Moniz MB, 1649-028 Lisboa, Portugal; Department of Cardiology, Unidade Local de Saúde de Santa Maria (ULSSM), CAML, CCUL@RISE, Faculdade de Medicina, Universidade de Lisboa, Avenida Professor Egas Moniz MB, 1649-028 Lisboa, Portugal; Department of Cardiology, Unidade Local de Saúde de Santa Maria (ULSSM), CAML, CCUL@RISE, Faculdade de Medicina, Universidade de Lisboa, Avenida Professor Egas Moniz MB, 1649-028 Lisboa, Portugal

**Keywords:** Oesophagopericardial fistula, Purulent pericarditis, *Streptococcus equi*, Effusive–constrictive pericarditis, Case report

## Abstract

**Background:**

Oesophagopericardial fistula is a rare but life-threatening condition that may lead to purulent pericarditis, which in turn carries a high risk of progression to constrictive pericardial disease.

**Case summary:**

A 50-year-old horse handler with advanced oesophageal carcinoma presented with acute chest pain, fever, and shock. Echocardiography showed a large pericardial effusion and pericardiocentesis yielded purulent fluid. *Streptococcus equi* was isolated. Computed tomography confirmed an oesophagopericardial fistula. The patient underwent percutaneous drainage, broad-spectrum antimicrobial therapy, and endoscopic stent placement. Despite initial improvement, he developed effusive–constrictive physiology and later refractory septic shock, leading to death.

**Discussion:**

This case highlights a rare zoonotic cause of purulent pericarditis and its progression to constrictive physiology despite appropriate management.

**Take-home messages:**

Early recognition, multidisciplinary management, and follow-up imaging are essential in complex pericardial infections.

## Graphical Abstract

**Graphical Abstract:**
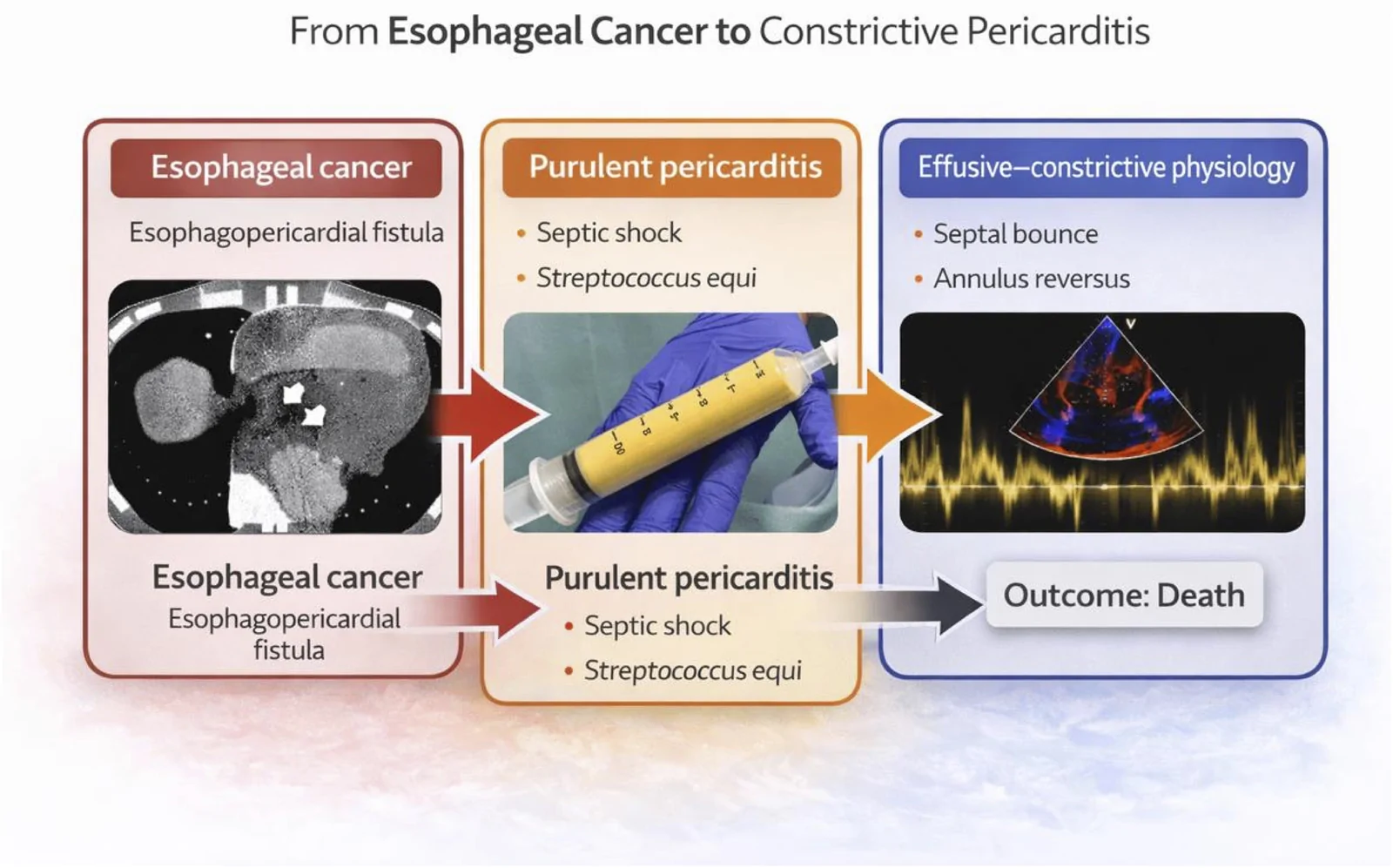
Oesophageal cancer complicated by oesophagopericardial fistula leading to purulent pericarditis due to *Streptococcus equi*, progressing to effusive–constrictive physiology and ultimately death.

Learning points

- Oesophagopericardial fistula is a rare but life-threatening cause of purulent pericarditis.
- Zoonotic pathogens such as *Streptococcus equi* should be considered in patients with relevant occupational exposure.
- Purulent pericarditis may evolve to effusive–constrictive physiology despite appropriate drainage and antimicrobial therapy.

## Introduction

Oesophagopericardial fistula is a rare and life-threatening condition characterized by an abnormal communication between the oesophagus and the pericardial space. It most commonly occurs as a complication of advanced oesophageal malignancy and is associated with high morbidity and mortality due to severe infectious complications, including purulent pericarditis.^1,2^

Purulent pericarditis, although uncommon in the antibiotic era, remains a medical emergency with rapid progression to cardiac tamponade, septic shock, and death if not promptly recognized and treated.^3,4^ According to the ESC Guidelines,^5^ early diagnosis and prompt pericardial drainage combined with antimicrobial therapy are essential to improve outcomes.

Zoonotic pathogens are rarely implicated in pericardial infections. *Streptococcus equi*, a β-haemolytic *streptococcus* primarily associated with equine disease, has only exceptionally been reported as a cause of invasive human infection, typically in individuals with close occupational exposure to horses.^6,7^

Despite appropriate management, persistent pericardial inflammation may lead to constrictive physiology, reflecting ongoing fibrosis and impaired ventricular filling, a condition associated with significant morbidity.^8^

We report a case of oesophagopericardial fistula complicating advanced oesophageal carcinoma, presenting as purulent pericarditis caused by *S. equi*, with subsequent progression to effusive–constrictive physiology.

## Summary figure

**Figure:**
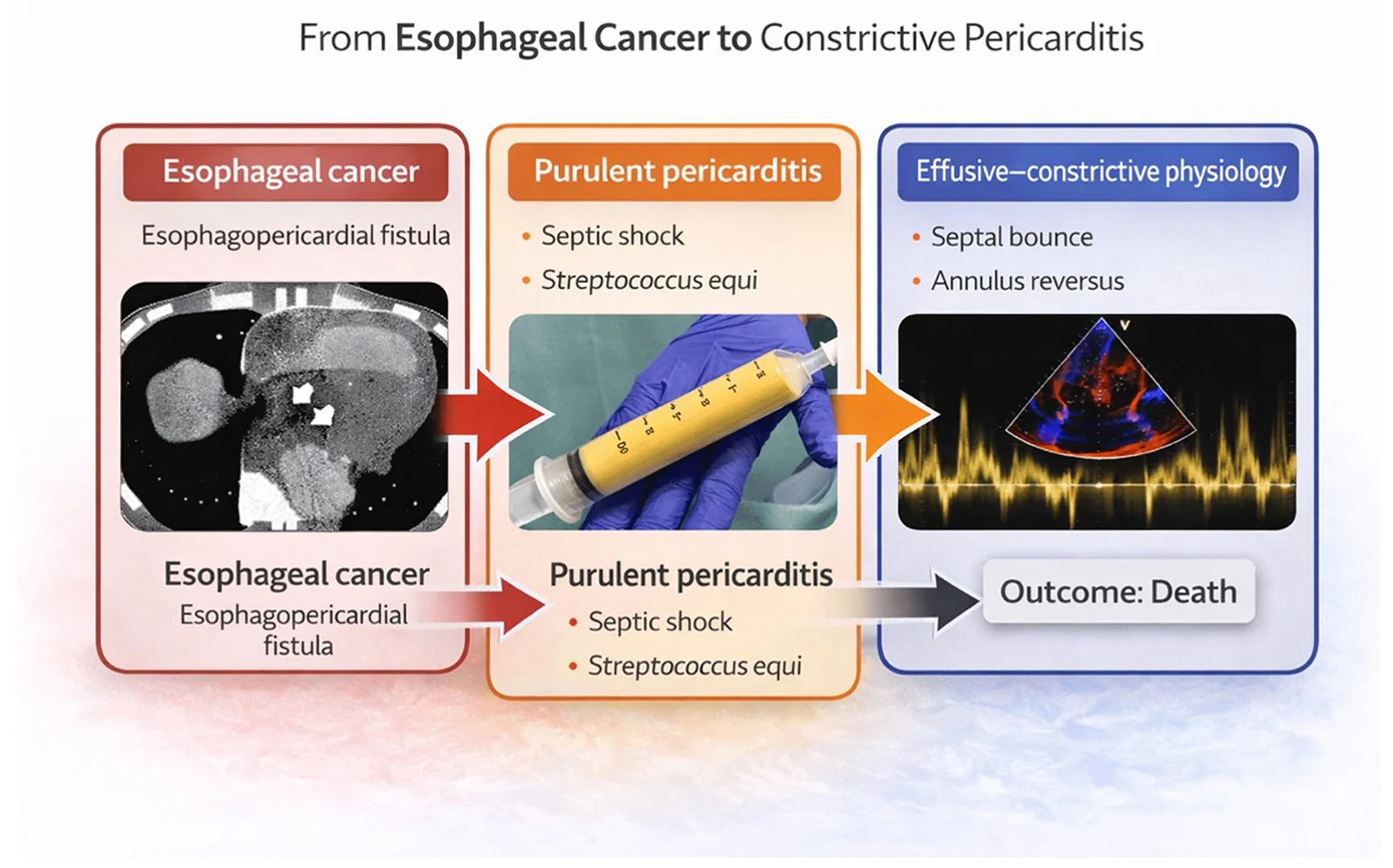


## Case presentation

A 50-year-old man who worked as a horse handler, with history of poorly differentiated squamous cell carcinoma of the mid-oesophagus with hepatic and nodal metastases (mediastinal and supraclavicular), for which systemic chemotherapy had been planned but not yet initiated, as well as active smoking (30 pack-years) and alcohol use disorder, presented with a 2-day history of rapidly progressive symptoms, including sudden-onset chest pain, fever, and chills. The pain was acute in onset and rapidly worsening. On presentation, he was haemodynamically unstable, with sinus tachycardia (142 beats/min) and hypotension (blood pressure 89/58 mmHg). Physical examination revealed jugular venous distension at 45° and muffled heart sounds.

Laboratory evaluation showed leukocytosis (29.1 × 10^9^/L; normal 3.6–10.5) with neutrophilia (87%; normal 40–70%), elevated C-reactive protein (30 mg/dL; normal <0.5), anaemia (haemoglobin 7.9 g/dL; normal 12.5–15.8), thrombocytosis (492 × 10^9^/L; normal 160–370), and coagulopathy (international normalized ratio 2.6).

Electrocardiography demonstrated sinus tachycardia (139 beats/min) without conduction abnormalities. Subtle diffuse concave ST-segment elevation and possible PR-segment depression were noted, suggestive of pericardial involvement **(*Figure 1*)**.

**Figure 1:**
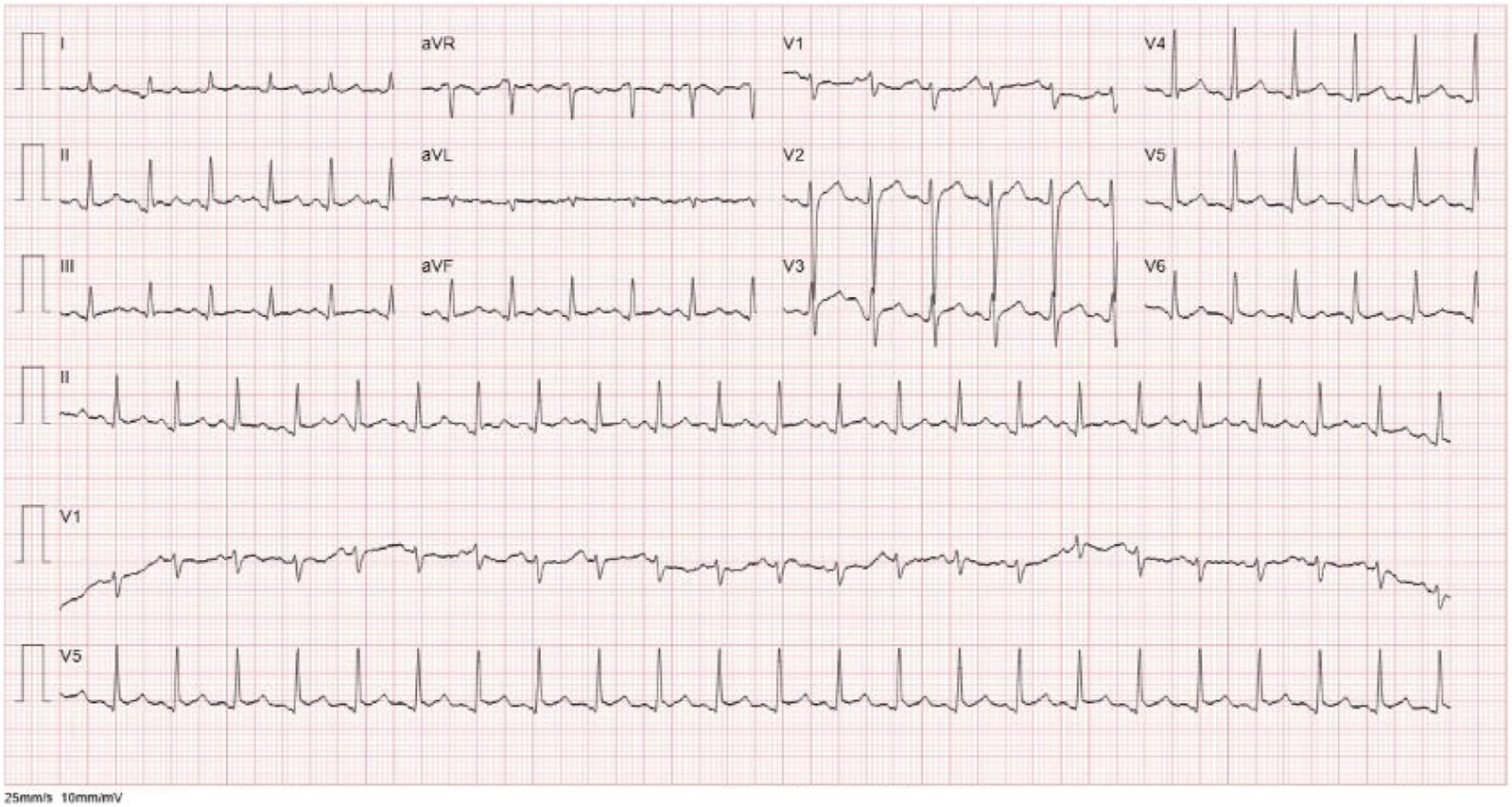
Electrocardiogram. Electrocardiogram at presentation showing sinus tachycardia (139 beats/min), subtle diffuse concave ST-segment elevation, and PR-segment depression, suggestive of pericardial involvement.

Transthoracic echocardiography demonstrated a large pericardial effusion with echogenic content, measuring up to 30 mm adjacent to the right ventricular free wall. Although there was no clear diastolic collapse of the right atrium or right ventricle, the inferior vena cava was dilated (26 mm), raising concern for elevated right-sided pressures in the setting of impending cardiac tamponade. Contrast-enhanced computed tomography demonstrated a lower oesophageal lesion with focal wall discontinuity and extraluminal gas associated with pneumomediastinum between the pulmonary artery trunk and the ascending aorta, highly suggestive of an oesophagopericardial fistula (***Figure 2***). There was an asymmetric pericardial effusion with pericardial thickening and enhancement (maximum thickness 24 mm), as well as a left pleural effusion. Additional findings included pulmonary and hepatic nodules and mediastinal lymphadenopathy, consistent with known metastatic disease.

**Figure 2:**
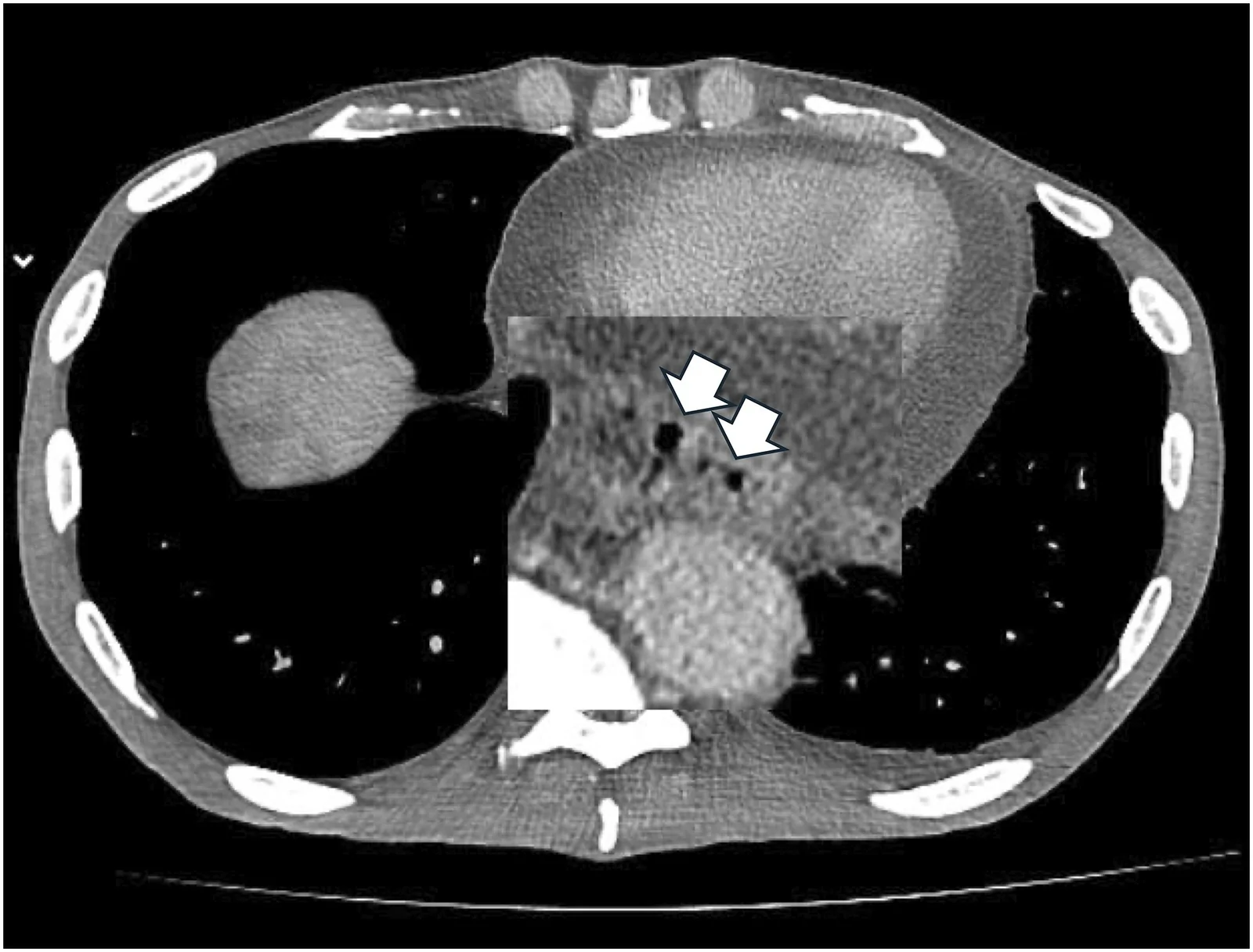
Contrast-enhanced computed tomography. There are small foci of extraluminal air (arrows) adjacent to the oesophagus and pericardium, with loss of normal fat planes, suggestive of an oesophagopericardial fistula. A moderate pericardial effusion is also present.

Urgent pericardiocentesis resulted in immediate drainage of approximately 500 mL of purulent fluid (***Figure 3***). Pericardial fluid analysis revealed a highly inflammatory exudate, with 178 771 nucleated cells (88% polymorphonuclear cells), glucose <2 mg/dL, elevated protein (5.5 g/dL), and markedly elevated lactate dehydrogenase (10 887 U/L), consistent with purulent pericarditis. Microbiological analysis identified *S. equi*, while concurrent blood cultures were negative. Surgical pericardial drainage was deferred due to the patient’s haemodynamic instability at presentation and a less invasive strategy with percutaneous drainage was initially pursued.

**Figure 3:**
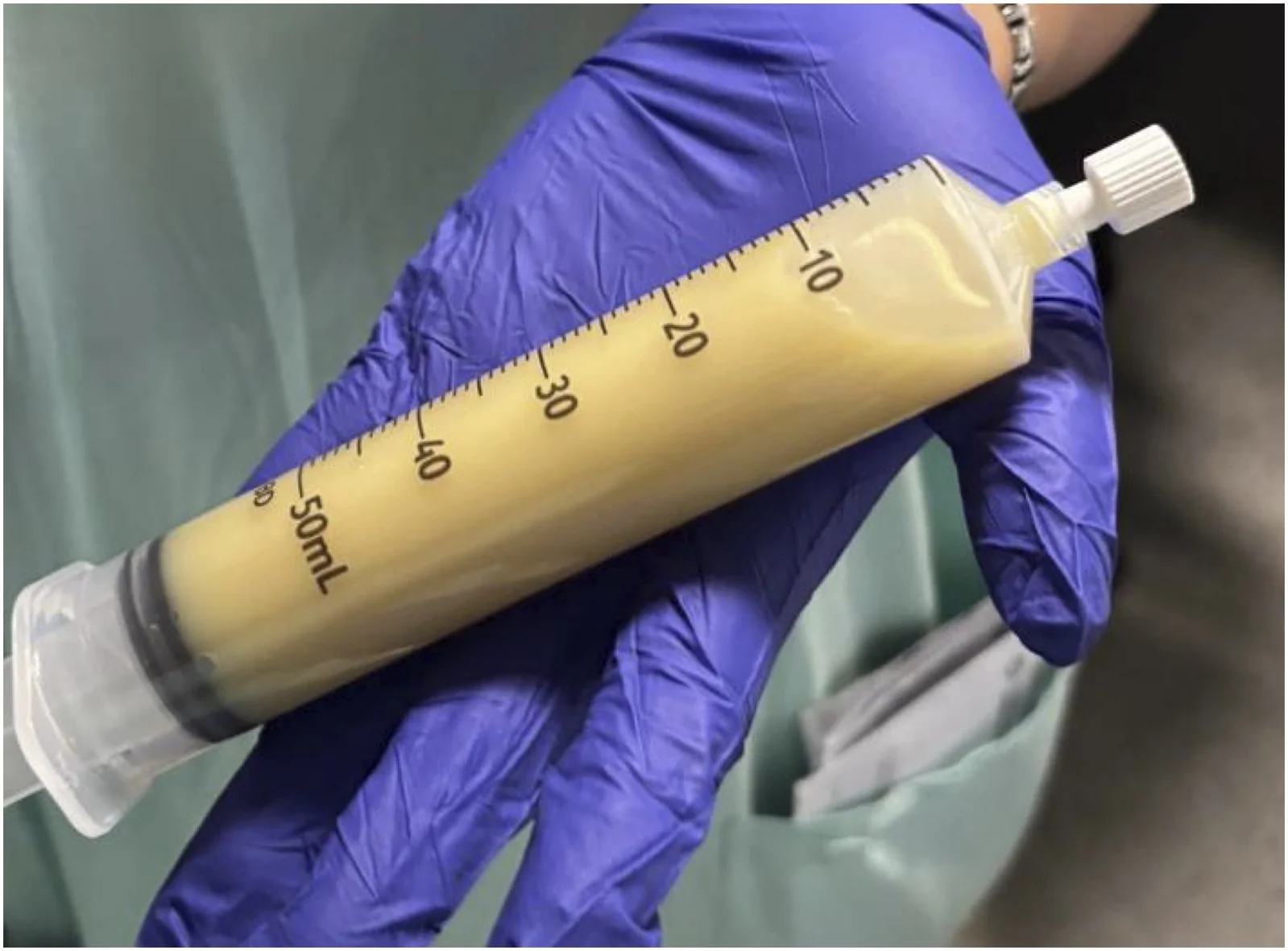
Purulent pericardial fluid. Thick, yellow turbid fluid obtained during pericardiocentesis, consistent with purulent pericarditis. Microbiological analysis identified *Streptococcus equi*.

*Streptococcus equi* was found to be sensitive to penicillin, but broad-spectrum antimicrobial therapy with meropenem and fluconazole was continued after discussion with the infectious diseases team, given the severity of presentation and the inability to exclude concomitant mediastinitis. Follow-up blood cultures obtained after initiation of antimicrobial therapy were negative.

To address the underlying structural cause, endoscopic closure of the oesophagopericardial fistula was performed with placement of a partially covered self-expanding metallic stent (WallFlex, 18 × 23 mm) under fluoroscopic guidance (***Figure 4***). Post-procedural imaging confirmed appropriate stent positioning with satisfactory intraluminal contrast progression and no evidence of leakage.

**Figure 4:**
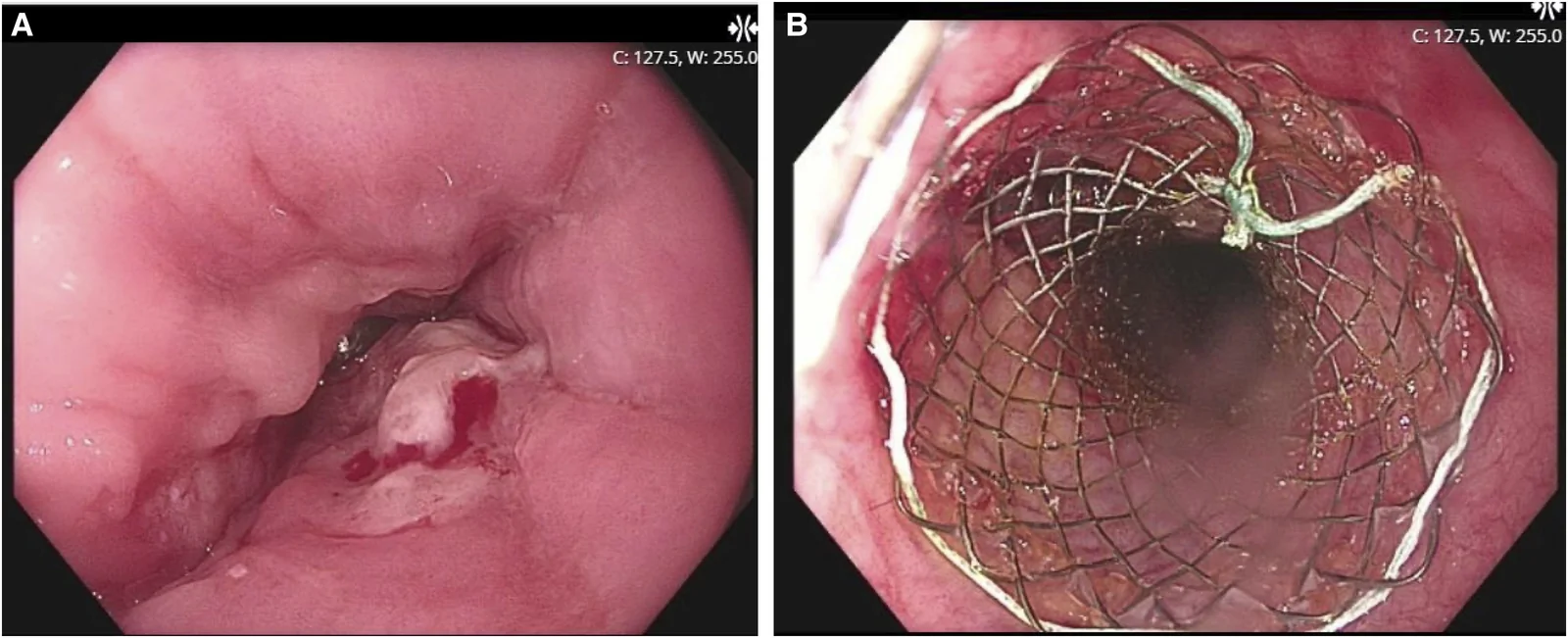
Endoscopic images before and after oesophageal stent placement. (*A*) Endoscopic appearance of the oesophageal lesion before intervention. (*B*) Endoscopic appearance following placement of the partially covered self-expanding metallic stent.

Following initial management, the patient showed significant clinical improvement, with resolution of haemodynamic instability and septic shock. He became afebrile and inflammatory markers progressively decreased (nadir C-reactive protein: 13.5 mg/dL). During hospitalization, however, the patient developed progressive signs of right-sided heart failure, including lower limb oedema, jugular venous distension, and ascites. Repeat transthoracic echocardiography demonstrated a thickened visceral pericardium with a small residual pericardial effusion, measuring 7 mm, predominantly posterior (see Supplementary material online, *Videos S1*–S3). Findings were suggestive of effusive–constrictive physiology, including septal bounce (***Figure 5****A*; Supplementary material online, video  *S1*), plethoric inferior vena cava, and *annulus reversus* (***Figure 5****B and C*), the latter interpreted as a supportive rather than an isolated diagnostic finding. Respiratory variation in mitral and tricuspid inflow velocities was 19% and 23%, respectively. Hepatic vein Doppler recordings were of insufficient quality for reliable assessment of expiratory diastolic flow reversal. Right ventricular systolic dysfunction was also noted [Tricuspid Annular Plane Systolic Excursion (TAPSE) 12 mm; S′ 8 cm/s], while left ventricular systolic function and valvular structure remained preserved. These findings were considered suggestive of effusive–constrictive physiology. On day 23 of hospitalization, the patient developed recurrent refractory septic shock. Repeat echocardiography showed no progression of pericardial effusion and similar features of constrictive physiology. Computed tomography confirmed appropriate positioning of the oesophageal stent, without evidence of leakage or findings suggestive of mediastinitis. Despite aggressive medical management, the patient’s condition deteriorated and he ultimately died.

**Figure 5:**
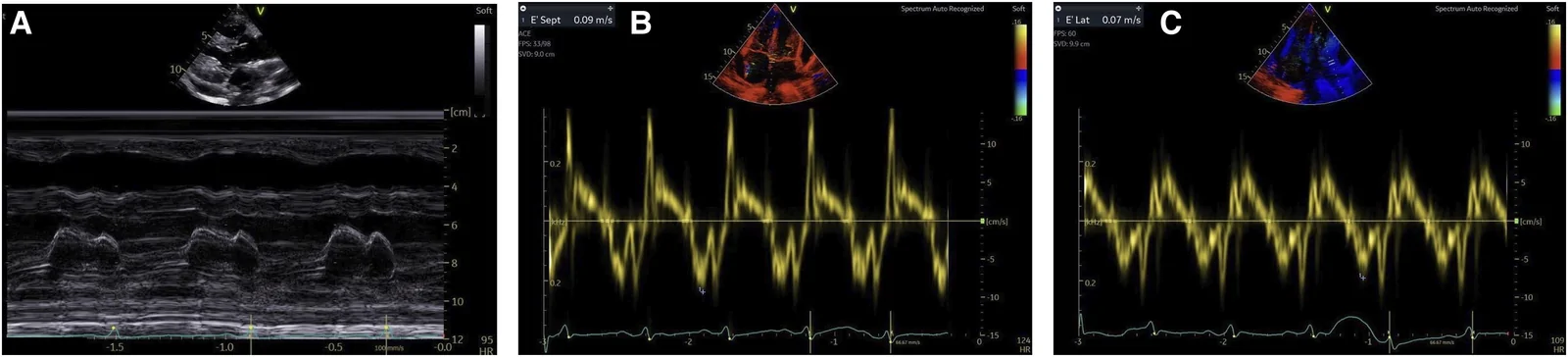
Transthoracic echocardiography. (*A*) M-mode imaging suggesting septal bounce. (*B*) Tissue Doppler imaging of the septal mitral annulus demonstrating preserved *e*′ velocity. (*C*) Tissue Doppler imaging of the lateral mitral annulus showing reduced *e*′ velocity. The combination of preserved septal and reduced lateral *e*′ velocities (*annulus reversus*) is supportive of constrictive physiology.

## Discussion

This case exemplifies a complex clinical scenario in which structural disruption and infection converge, resulting in severe inflammatory pericardial disease. The coexistence of an oesophagopericardial fistula and purulent pericarditis is a rare but recognized complication of advanced oesophageal malignancy.^1,2^ Other causes of oesophagopericardial fistula are surgical procedures and radiofrequency atrial fibrillation ablation.^9^

A key diagnostic challenge in this case was distinguishing between malignant and infectious pericardial involvement in a patient with known oesophageal carcinoma. The acute clinical presentation, characterized by fever, rapid haemodynamic deterioration, and markedly elevated inflammatory markers, favoured an infectious aetiology, consistent with previous descriptions of purulent pericarditis.^3,4^ Multimodality imaging played a central role in establishing the diagnosis and guiding management.

The identification of *S. equi* represents a rare but clinically relevant finding. Although primarily associated with equine infections, this zoonotic pathogen may cause invasive disease in humans with occupational exposure, highlighting the importance of a detailed exposure history in atypical infections.

Despite initial clinical improvement following pericardial drainage and antimicrobial therapy, the patient developed features consistent with effusive–constrictive physiology.^8^ This evolution likely reflects persistent pericardial inflammation leading to fibrosis and impaired ventricular filling. Current ESC Guidelines^5^ emphasize the importance of close clinical and imaging follow-up in patients with complicated pericardial disease, as progression to constriction may occur despite appropriate initial management.

Management of oesophagopericardial fistula requires a multidisciplinary approach. Endoscopic stent placement has emerged as a minimally invasive strategy for achieving fistula closure in selected patients, particularly when surgical intervention is not feasible due to clinical instability or advanced disease.^1,2^ Despite adequate source control and antimicrobial therapy, the patient developed refractory septic shock and died, highlighting the high mortality associated with this condition.

This case underscores the complex interplay between structural disease, infection, and inflammation, highlighting the need for early recognition, multidisciplinary management, and vigilant follow-up to detect potentially fatal complications.

## Supplementary Material

ytag700_Supplementary_Data

## Data Availability

The data underlying this article will be shared on reasonable request to the corresponding author.

## Lead author biography

Dr. Sofia Esteves is a 28-year-old cardiology resident in her fourth year of training. She has a particular interest in clinical cardiology and cardiovascular imaging, with a focus on the integration of multimodality imaging into patient management. Her work is centred on the diagnosis and treatment of complex cardiovascular conditions, combining clinical assessment with advanced imaging techniques.

## Notes

**Consent:** The authors confirm that written consent for submission and publication of this case report, including images and associated text, has been obtained from the patient, in line with COPE guidance.
